# Comparison of bioactive compounds and health promoting properties of fruits and leaves of apple, pear and quince

**DOI:** 10.1038/s41598-021-99293-x

**Published:** 2021-10-12

**Authors:** Aneta Wojdyło, Paulina Nowicka, Igor Piotr Turkiewicz, Karolina Tkacz, Francisca Hernandez

**Affiliations:** 1 Department of Fruit, Vegetable and Nutraceutical Plant Technology, Wrocław University of Environmental and Life Sciences, 37 Chełmońskiego Street, 51-630 Wrocław, Poland; 2 Department of Plant Science and Microbiology, Universidad Miguel Hernández de Elche, Carretera de Beniel, km 3.2, Orihuela, 03312 Alicante, Spain

**Keywords:** Secondary metabolism, Obesity, Metabolic disorders, Neurological disorders, Glycosides, Monosaccharides, Biochemistry, Plant sciences, Environmental sciences, Diseases

## Abstract

This paper presents characterization of healthy potential new sources of functional constituents with reference to basic plant sources. In this study, the phenolics, triterpene, isoprenoids (chlorophylls and carotenoids), amino acids, minerals, sugars and organic acids of different cultivars of pome species—apple, pear, quince—leaves vs. fruits and their enzymatic in vitro enzyme inhibition of hyperglycemic (α-glucosidase, α-amylase), obesity (pancreatic lipase), cholinesterase (acetylcholinesterase, butylcholinesterase), inflammatory (15-LOX, COX-1 and -2) and antioxidant capacity (ORAC, FRAP, ABTS) were evaluated. Leaves of pome species as a new plant sources were characterized by higher content of bioactive and nutritional compounds than basic fruits. The dominant fraction for quince, pear, and apple fruits was polymeric procyanidins. In quince and pear leaves flavan-3-ols, and in apple dihydrochalcones dominated. Triterpene was present in equal content in leaves and fruits. Leaves are excellent sources of amino acids and minerals (especially Ca, Mg, Fe, and K), with high content of organic acids and low content of sugars compared to fruits of pome species. Leaves of apples and pears most effectively inhibited COX-1, COX-2, α-amylase, and α-glucosidase enzyme but quince leaves showed the most effective inhibition of pancreatic lipase, AChE and BuChE, 15-LOX, and antioxidant capacity, which particularly correlated with bioactive compounds. Present study shows that leaves are promising sources of valuable compounds and may be used to produce functional foods as well as for medical purposes.

## Introduction

The medicinal value of different plants derives from and correlates with some biochemical substances that produce a definite physiological action in the human body. Numerous investigations have proven that plants are rich in biologically active compounds that exhibit beneficial health-promoting properties. Fruits and vegetables are basic plants used every day in the diet worldwide, but researchers still focus on new sources of bioactive compounds with high biological properties.

The study of new potential sources of food and the bioactive component in the context of health promotion through the diet has been and will continue to be an important research field, especially when nowadays the world population is growing^1^.

It is widely recognized that pome species are among the most popular fruits and rich in bioactive compounds. In the world, the annual harvest of apple, pear, and quinces totals several million tonnes and as a side stream of fruit production, large quantities of leaves are produced every year. It is well known that eating “an apple a day keeps the doctor away”, and pears or quinces brings many health benefits^2–9^. Whereas these fruits are important food for human consumption and raw materials for the food industry, the leaves are left unused as agricultural waste and only a small fraction of these leaves are processed for use in certain food products.

As mentioned previously^10^ leaves of some edible fruits are one of the promising sources of bioactive compounds with potential health properties. Extracts of leaves e.g. Passiflora^11^, currant^12^ walnut^13^, raspberry^14^, sea buckthorn^15^, eggplant^16^ or mulberry^17^ are used in folk and traditional medicine (e.g. Chinese, Tibetian, Turkish, Mongolian, Native Americans) to chronic diseases as treat diabetes, hypolipidemic, hypertension, digestive disorders, reducing blood glucose, cardiovascular and skin diseases, stroke, anxiety, irritability, migraines, insomnia, opiate withdrawal, and many other.

Until now research was focused basically on bioactive compounds such as polyphenolic compounds^2,18,19^. Previously research showed that apple leaves are rich in polyphenolic compounds (about 160.65 g/kg dm), especially phloretin-2’-glucose and procyanidins^20^. Similarly, quince leaves are rich in polyphenolic compounds, ranging from 4.9 to 16.5 g/kg dm, especially kaempferol derivatives^2^. Polyphenolics scavenge free radicals and inhibit their production, and their action is additionally associated with other biological properties such as anti-inflammatory, anti-microbial, anti-cancer, cardiovascular and other activity. Consumption of some leaves as donors of bioactive compounds is highly popular in Asian countries such as Japan, Korea, and Vietnam^17^. Recently the European Medicines Agency approved the circulation of leaf infusions and extracts of *Arctostaphylos uva-ursi, Rubus idea* and *Ribes nigrum* as herbal medicinal products based on their traditional uses. Thus, leaves are potential new raw materials for the production of nutraceuticals and functional ingredients of food. However, in literature still limited information is presented about the chemical composition, isoprenoids, triterpenes or amino acids, or even sugars and organic acids. Additionally, to our knowledge, little information is available on the influence of these compounds on hyperglycemic, obesity, lipoxygenase, cholinesterase or anti-inflammatory enzyme activity.

Therefore the novelity and for a better understanding of potential new sources of bioactive compounds of leaves of apple, pear, and quince it is important in this context to investigate the contents of some bioactive and nutritional compounds and their enzymatic in vitro inhibitory activity against hyperglycemic (α-glucosidase, and α-amylase), obesity (pancreatic lipase), lipoxygenase, cholinesterase (acetylcholinesterase and butylcholinesterase), inflammatory (cyclooxygenase 1 and 2) and antioxidant capacity (ORAC, FRAP, ABTS). Additionally, the variation in the content and profile of polyphenols, isoprenoids, triterpenes, amino acid and other chemical properties in apple, pear, and quince leaves were investigated as a function of the cultivar. Leaves of apple, pears and quince have not been thoroughly characterized to date. The aim of this paper is to verify research hypotheses assuming that pome fruit (apple, pears and quince) tree leaves are as a new, unconventional and valuable source of nutritional and bioactive compounds vs. fruits. Hence, the search for new sources of natural antioxidants is currently of major interest to scientists.

## Results and discussion

### Chemical composition of apple, pear and quince fruits and leaves

#### Organic acid, sugars and mineral components

**Figure 1:**
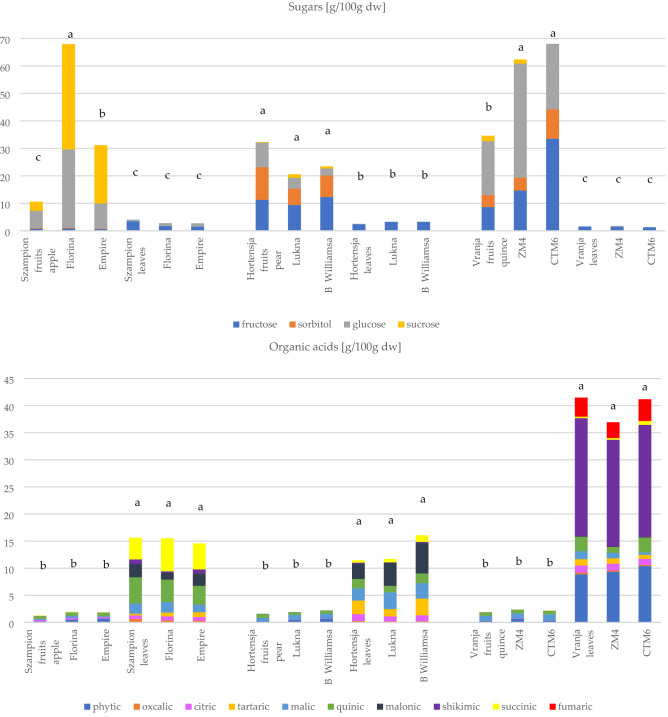

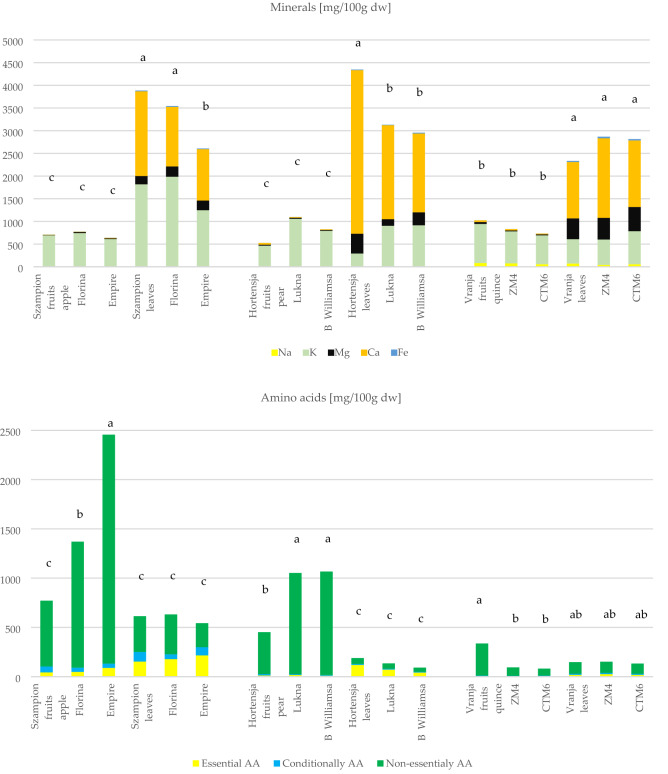
Total content of sugars and organic acids [g/100 g dw], minerals and amino acids [mg/100 g dw] in fruits and leaves of some selected species and cultivars. Mean of 3 replications ± standard deviation followed by the same letter were significantly different (p < 0.05) according to Tukey’s least significant differences test; Letters a,b,c,d, represent significance in content (p < 0.05).

The taste of fruits provides very important stimuli for the receptors, and thus play an important role in consumers’ determination of food quality. Sugars and organic acids are the major components responsible for this attractive taste and freshness. As presented in Fig. 1, it was noted that sugars were dominant in fruits but organic acids in leaves, and the differences between fruits and leaves were significant at *p* < 0.001.

Free sugar levels determined by HPLC-ELSD were from 10.6 to 68.0 g/100 g dry weight (dw) for fruits and from 1.3 to 4.0 g/100 g dw for leaves. Species (apple × pear × quince), part of plant (fruits x leaves) and cultivar had a significant influence (p < 0.05) on the content of sugars. The content of sugars in fruits was similar to other results presented previously^21^. As in many other fruits, glucose and fructose were dominant and represented approx. 75% of all sugars. In leaves it was noted that fructose was the primary sugar in all samples, but glucose was found only in trace amounts in some cultivars. Trace amounts of sorbitol were found in fruits, but pome fruits were not an excellent source of this compound. Sorbitol is synthesized in the leaves from glucose-6-phosphate by sorbitol-6-phosphate dehydrogenase enzyme and afterward is translocated to the fruits. Therefore, absence of this sugar in leaves means that biochemical conversation occurred completely.

Phytic, oxalic, citric, tartaric, malic, quinic, malonic, shikimic, succinic and fumaric acids were measured in fruits and leaves (Fig. 1). Total organic acid levels varied from 1.3 to 2.4 g/100 g dw for fruits and from 11.5 to 41.5 g/100 g dw for leaves. The profile and content of organic acids are confirmed by other studies. In fruits the dominant acids were: malic > quinic and citric acids, whereas in leaves they were quinic, malic, malonic and tartaric acids, but the content was significantly dependent on species and cultivar. Additionally, quince fruits and leaves were richer in phytic and fumaric acids than other evaluated pome fruits (*p* < 0.05). The rest of organic acids together accounted for < 2% of total or were not found in detectable amounts. The content of individual oxalic and shikimic acids (*p* < 0.05) differed widely among the cultivars. Differences in amount of organic acids in pome species may be due to local climate and soil conditions and altitude, as well as genetic factors^22^. Organic acids are important for the human and animal body because they can positively influence the microflora in the gastrointestinal tract, thus improving nutritional uptake and health.

The sugar/acid ratio is an important index for estimation of the organoleptic properties and consumer’s acceptability for taste. For the investigated fruits this ratio varied from 8.4 to 35.5 whereas for leaves it was < 0.5. *Chaenomeles* fruits are characterized by extremely low content of sugars (< 3.98 g/100 g fresh weight [fw]) but high content (51.5 to 110.3 g/kg fw) of organic acids, and with a ratio of 0.1 to 0.6^23^.

#### Mineral content

Throughout the world, there is increasing interest in the role of dietary minerals in relation to the prevention of several diseases^22^. In the analyzed fruits and leaves five minerals were quantified but only three are major macro-elements: potassium, calcium, magnesium. In general, sodium and ferrous levels were low. Their total contents were significantly different between fruits and leaves (p < 0.05; Fig. 1) but not between cultivars within type of pome species. Compared to fruits, leaves of all pome species are significant sources of Ca, Mg, Fe and K. It was noted that content of minerals in leaves was 3–6 times higher than in fruits, especially K and Ca content. The greatest differences (*p* < 0.05) between leaves and fruits were noted for Fe content, particularly quince leaves. This is very valuable information, especially for people with high iron requirements (i.e. pregnant women, child-bearing age)^24^.

Mg was the most abundant macro-element for quince leaves and fruit cultivars, 457.3–534.0 mg and 20.3–41.9 mg/100 g dw, respectively. Only quince fruits and leaves showed high content of Na, contrary to apple and pears fruits and leaves (< 7 mg/100 g dw). This fact could be of interest to people with special low sodium diets because nowadays salt is available everywhere. Among the foods evaluated by Leterme et al.^25^, leaves are unconventional sources of minerals and appear as outstanding mineral sources, which have the highest contents of Ca, Mg, S, Fe, Mn and Cu. The leaves of *Trichanthera* have average Ca content of 6.2 g/100 g dw (Leterme et al., 2006). Other unconventional sources of minerals are peels of fruits (pomelo, orange, lemon, mandarin), i.e. K, Ca and Mg^24^.

It is known that fruits of pome species have high nutritional properties but leaves should be classified as a ‘source of minerals’. The differences in content of minerals may also be ascribed to different species, fruit maturity, agricultural practice, and ecological conditions, such as climate, altitude, soil fertility and seasonal variations^22^. Micronutrients are involved in numerous biochemical processes and an adequate intake of certain micronutrients relates to the prevention of deficiency diseases. Phosphorus, together with Ca, participates in the formation of strong bones and teeth^24,25^. The daily requirements of an adult man are as follows (mg/d): 10–15 Fe, 300–400 Mg, 700–800 P, 800–1200 Ca, 500 Na, 12–15 Zn, 2–3 Cu (FAO).

#### Amino acids

The concentrations of free amino acids present in apple, pears, and quince fruits vs. leaves are shown in Fig. 1. Apple leaves and fruits are characterized by significantly higher content of amino acids than pears and quince samples. All pome fruits have significantly higher content of amino acids than leaves, and cultivars were also differentiated by their content. As previously mentioned by Mykhailenko et al.^26^ the above ground organs (i.e. leaves, flowers) have a higher content and more diverse composition of amino acids than their underground organs (i.e. corms and rhizomes).

Nineteen amino acids were determined in pome species and they were categorized into essential (histidine, isoleucine, leucine, lysine, phenylalanine, threonine, tryptophan), conditionally essential (arginine, glutamine, glycine, proline, tyrosine), and non-essential (alanine, asparagine, aspartic acid, cysteine, glutamic acid, serine, alanine) amino acids^27,28^. Methionine and valine were absent. As a general rule, the non-essential fraction was significantly dominant in all fruits and leaves, besides apple leaves where essential and non-essential fractions were equal. Leaves of apple, pear, and quince had higher content of essential amino acids while for fruits this fraction was marginal. The content of essential amino acids for people is important because they are synthesized only by plants and people are still looking for new rich sources of these type amino acids.

The pome leaves had a more diverse amino acid composition compared to pome fruits (Fig. 1). In apple and pear fruits and leaves aspartic acid was dominant but additionally in fruits *O*-phospho-l-serine, and in pear leaves additionally glutamic acid and cysteine content was observed. Meanwhile in the fruit of quince aspartic acid dominated, but in leaves cysteine, aspartic and glutamic acid dominated. Generally, the amino acid profile is similar as previously reported in the literature^27^. For comparison, the sum of amino acids ranged from approx. 0.03 to 0.14 and 0.05 to 0.18 mg/100 g for quince pulps and peels, respectively^27^. It seems that apple has some similarities with quince and pear; asparagine and aspartic acid are usually two of the major free amino acids in these pome species^27^. Compared to results present by Turkiewicz et al.^29^ apple, pear and quince fruits and leaves are characterized by lower content of amino acids than some cultivars of flowering quince fruits (between 15.87 and 2326.33 mg/100 g dw), but they present similar content to a different species of rosehip^28^ or sprouts and microgreens^30^.

Aspartic acid is synthesized by direct amination but alanine and glutamic acid are formed as a result of reductive amination. All other amino acids are secondary ones, because they are formed as a result of transamination of the amino acids listed above with the corresponding keto acids that arise during the metabolism, as well as by the conversion of some acids to others^26,28^. Changes in amino acid contents in different plants depend on numerous factors such as source of nutrition, nutritional requirement, especially nitrogen fertilization, biotic and abiotic factors. Apart from nutritional properties, amino acids also affect taste and flavor, as a number of them have a distinctively bitter taste (e.g. tyrosine, arginine, leucine, valine, methionine, and histidine)^26–28^ Additionally amino acids exhibit different actions benefiting human health such as preventing cardiovascular disease, improving digestion, protection from arteriosclerosis and diabetes mellitus, etc.^28^.

#### Polyphenols

As shown in Table 1, among the investigated sample, species, cultivar, and fruits vs. leaves had a significant influence of the content of polyphenols. Significantly higher concentration of polyphenols was noted for leaves (approx. 10,825.9 mg/100 g dw) than in fruits (approx. 3275.0 mg/100 g dw). Higher content of polyphenols was noted for quince fruits and leaves (approx. 9128.2 mg/100 g dw) than for apple (approx. 7289.0 mg/100 g dw) and pears (approx. 5904.8 mg/100 g dw). Cultivar had a significant influence on content of polyphenols except monomers of flavan-3-ols and phenolic acids. Leaves turned out to be richer in phenolics than fruits due to the complexity of the biosynthesis process in plants, which is dependent on various factors, including place of cultivation, environmental conditions, drought resistance, frost hardiness, biotic and abiotic stress^31,32^. As reported by Jaakola et al.^31^, a higher content of polyphenolic compounds was identified in leaves of plants growing under intensive sunlight, which results in enhanced gene expression coupled with phenolic biosynthesis.

**Table 1:** Comparison of polyphenols [mg/100 g dw] content in fruits and leaves of some selected species and cultivars of apple, pear, quince fruits and leaves.

Species	Cultivars	Flavan-3-ols		Dihydrochalcones	Phenolic acid	Flavonols	Anthocyanins	Total
		PP	Monomers					
Apple	**Fruits**							
	Szampion	1325.0 ± 25.3	248.8 ± 9.5	12.0 ± 1.4	1117.1 ± 32.1	44.6 ± 4.6	1.9 ± 0.2	2749.4
	Florina	705.1 ± 12.6	261.9 ± 6.3	10.9 ± 1.1	60.1 ± 24.5	43.3 ± 2.4	0.9 ± 0.1	1082.2
	Empire	455.2 ± 11.2	145.8 ± 8.5	11.5 ± 1.1	36.3 ± 25.9	34.0 ± 3.6	0.9 ± 0.2	683.6
	**Leaves**							
	Szampion	622.7 ± 15.1	1320.3 ± 12.8	3520.1 ± 12.4	92.3 ± 8.5	3455.4 ± 12.5	7.0 ± 0.6	9017.7
	Florina	1032.7 ± 19.4	944.3 ± 15.6	3155.7 ± 21.2	181.5 ± 6.9	2360.1 ± 25.7	0.0 ± 0.0	7674.3
	Empire	1435.2 ± 23.6	861.7 ± 21.7	3552.7 ± 12.5	191.7 ± 10.5	1632.2 ± 27.1	0.0 ± 0.0	7673.5
Pear	**Fruits**							
	Hortensja	352.4 ± 12.2	197.7 ± 1.6	nd	105.6 ± 2.5	66.7 ± 2.4	0.0 ± 0.0	722.4
	Lukna	1069.8 ± 8.9	289.2 ± 3.7	nd	28.3 ± 1.1	66.0 ± 4.9	0.0 ± 0.0	1453.4
	Bonkreta Williamsa	728.6 ± 2.5	292.2 ± 5.7	nd	105.6 ± 2.8	21.6 ± 1.1	0.0 ± 0.0	1148.0
	**Leaves**							
	Hortensja	1328.9 ± 6.8	2327.0 ± 4.7	nd	3764.6 ± 3.7	2593.3 ± 6.9	0.0 ± 0.0	10,013.8
	Lukna	2126.2 ± 5.9	2955.6 ± 2.8	nd	2004.7 ± 9.7	5108.9 ± 15.9	1.4 ± 0.2	12,196.7
	Bonkreta Williamsa	2034.6 ± 8.9	2351.0 ± 3.2	nd	3604.6 ± 13.7	2393.5 ± 21.3	2.4 ± 0.2	10,386.1
Quince	**Fruits**							
	Vranja	5466.7 ± 14.4	431.0 ± 11.8	nd	309.7 ± 21.5	117.6 ± 4.6	nd	6325.1
	ZM4	4261.4 ± 16.4	287.3 ± 14.2	nd	414.7 ± 14.7	102.6 ± 3.8	nd	5066.0
	CTM6	5493.9 ± 18.6	249.0 ± 11.7	nd	316.3 ± 11.8	237.3 ± 6.9	nd	6296.5
	**Leaves**							
	Vranja	8639.4 ± 21.5	2394.1 ± 21.5	nd	251.9 ± 10.6	1822.0 ± 21.7	nd	13,107.3
	ZM4	5917.2 ± 26.8	1432.4 ± 12.5	nd	498.7 ± 9.6	2310.6 ± 25.7	nd	10,158.8
	CTM6	9919.0 ± 13.4	1467.1 ± 11.6	nd	444.2 ± 12.1	1653.2 ± 19.7	nd	13,483.5
Species	Apple	1984.6b	976.8b	1620.3a	701.0b	1769.7a	1.5a	5904.8b
	Pear	2495.7b	1692.9a	nd	2429.5a	1774.6a	2.2a	7289.0b
	Quince	6190.9a	1232.7ab	nd	1035.8b	1774.6a	0.0b	9128.2a
Fruits/leaves	Fruits	2534.4b	1926.8a	3.8b	618.1b	316.9b	0.8b	3275.0b
	leaves	3995.9a	415.2b	1132.7a	1563.9a	2819.2a	1.6a	10,825.9a
Cultivars	Szampion	3461.2bc	1514.1a	1658.0a	2226.9a	2393.2ab	5.6a	8627.1abc
	Florina	2849.0c	1107.6a	950.8a	1227.0a	1489.9b	1.0b	6448.4bc
	Empire	3432.6bc	1234.3a	1674.0a	1737.8a	1479.4b	1.6b	6927.8c
	Hortensja	2817.4c	1274.2a	0.0b	1824.4a	1968.7b	0.5b	6729.1c
	Lukna	3572.2bc	1633.1a	0.0b	908.8a	3222.0a	1.2b	8181.3abc
	Williamsa	2849.0c	1107.6a	0.0b	1227.0a	1489.9b	1.0b	6448.4bc
	Vranja	5313.8ab	1884.1a	0.0b	1569.3a	1610.7b	0.0b	9223.6ab
	ZM4	2849.0c	1107.6a	0.0b	1227.0a	1489.9b	0.0b	6448.4bc
	CTM6	5965a	1331.5a	0.0b	1668.5a	1586.3b	0.0b	9306.8a

Mean of 3 replications ± standard deviation followed by the same letter, within the same column were significantly different (p < 0.05) according to Tukey’s least significant differences test. Letters a,b,c,d, represent significance in content (p < 0.05).

*PP* polymeric procyanidins, *nd* not detected.

The profile and content of polyphenols were quite diverse and strongly dependent on the presence in different fruits and leaves according to the morphological part tested. In quince, pear and apple fruits procyanidins were the dominant fraction of polyphenols. In quince leaves were richer in flavan-3-ols than flavonols, in pear there was dominance of flavan-3-ols >  > flavonols > phenolic acids, and finally in apple the major content was dihydrochalcones > flavonols ~ flavan-3-ols. These results are similar to data previously mentioned in the literature^20,21,33^.

Flavan-3-ols were quantified as one of the predominant phenolics in the analyzed samples. These compounds present different properties, and beside them, they have antioxidative and antiproliferative properties. In addition, they also play a crucial role in shaping the astringent taste of foods. The highest content of these compounds was recorded in all quince and pear cultivars of fruits and leaves. A lower content was noted for apple fruits and leaves. Additionally, the analyzed cultivars of fruits and leaves exhibited large differences in content of flavan-3-ols, especially the cultivar Szampion, where fruits were noted to be richer than leaves. Szampion is a scab-resistant cultivar and, as previously reported, it accumulates significantly higher amounts of flavonoids, mainly flavan-3-ol compounds^34^.

Flavonols were the next most abundant phenolic group evaluated in leaves but not in fruits. Pear leaves were characterized by significantly higher content of flavonols than apple and quince, but the largest differences between fruits and leaves were noted for pear and apple. Leaves of pome species are interesting sources of flavonol compounds. This difference in content results from the fact that these compounds are mainly located in the top layer of plants, protecting them from harmful UV-A and UV-B radiation. Previous studies reported that flavonols (especially moieties of quercetin or kaempferol) accumulate in higher amounts in response to increased UV-B radiation^32^. Shading of the fruits (flavonols in fruits mainly located in skin) during development has been found to reduce the accumulation of flavonols and to inhibit the transcription of the corresponding flavonoid pathway genes^31,32^. Jaakola et al.^31^ discovered that the content of flavonol derivatives in sun-exposed leaves was three times higher than in shaded leaves. The content of flavonols in the daily diet, which can vary between 5 and 40 mg/day^35^, is important because they reduce the incidence of cardiovascular diseases and inhibit cancer cell growth^35^. High concentration of flavonol glycosides in apple leaves was noted earlier^20^. As previously reported^35^, among the richest sources of flavonols are onions (2.5–6.5 g/g), sea buckthorn (0.9 g/100 g dw^36^; and cranberry (1.2 g/100 g dm)^10^.

Phenolic acids were the next abundant group evaluated in leaves and fruit. Only pear leaves were characterized by a higher content of phenolic acid than fruits. Similar results were presented previously by Kolniak et al.^37^. Differences between leaves and fruits for quince were marginal, but for apple, the trend was not obvious because the content of phenolic acid was strongly dependent on cultivar (Table 1).

Another important class of naturally occurring flavonoids is dihydrochalcones, found only in apple leaves and fruits. These observations have also been confirmed previously^20^. Their content ranged from 3155.7 to 3552.7 mg/100 g dw for leaves and 10.9 to 12.0 mg/100 g dw for fruits. Florina cv. had the lowest content of dihydrochalcones compared to Szampion and Empire cultivars Mikulic-Petkovsek et al.^34^ reported that apple leaves exhibited low content of chlorogenic acid (0.0–1.0 mg/g dw) and procyanidin (0.5–0.9 mg/g dw) while the concentration of phloridzin ranged between 70 and 115 mg/g dw. Plants containing chalcones have been employed in traditional herbal medicine for centuries^35^. For that reason, the recent results of research present a wide spectrum of biological activities including antioxidative, antibacterial, anti-inflammatory by inhibiting COX-1 and COX-2 activity, anticancer against prostate cancer cells, and immunosuppressive potentials^35^.

Anthocyanin contents in fruits are marginal and for apple fruits the level was > 2 mg/100 g dw. In leaves, the composition of anthocyanins was more diverse and the concentrations were considerably higher, even tenfold, than in the fruits (Table 1). These compounds are strongly UV-absorbing, and accumulate in leaves mainly in the epidermal cells of the plant tissues^31,32^. Higher accumulation of anthocyanins and their photoprotective role have been observed in various species after UV-B- or UV-A-induced lights^32^, e.g. for cranberry leaves, where leaves are red^31^. Additionally, anthocyanins are compounds which show a number of health-promoting properties. In vitro and in vivo research trials have demonstrated anthocyanidins as compounds with biological activity, but this effectiveness also depends on the amount in a given plant^35^.

#### Isoprenoid

Carotenoids are associated with chlorophylls in the photosynthetic apparatuses of plants, and play a crucial role protect against photooxidation. The carotenoid and chlorophyll contents, presented in Table 2, significantly (p < 0.05) varied depending on the (*i*) species, (*ii*) morphological part (leaves vs. fruits) and (*iii*) cultivars.

**Table 2:** Comparison of isoprenoids (carotenoid and chlorophylls; mg/100 g dw) content in fruits and leaves of some selected species and cultivars of apple, pear, quince fruits and leaves.

Species	Cultivars	9-*cis* or 9-*cis'*-lutein	Σ β- and 9-*cis-*β-carotene	Chlorophylls				Pheophytin				Total
				*b*	*b’*	*a*	*a'*	*b*	*b’*	*a*	*a’*	
Apple	**Fruits**											
	Szampion	4.8 ± 0.2	3.0 ± 0.3	nd	nd	nd	nd	nd	nd	4.9 ± 0.2	0.0 ± 0.0	12.6
	Florina	2.1 ± 0.1	1.5 ± 0.1	nd	nd	nd	nd	nd	nd	2.0 ± 0.1	0.0 ± 0.0	5.6
	Empire	3.1 ± 0.1	1.4 ± 0.2	nd	nd	nd	nd	nd	nd	2.6 ± 0.2	0.0 ± 0.0	7.0
	**Leaves**											
	Szampion	133.4 ± 2.6	73.2 ± 2.7	17.6 ± 0.9	2.9 ± 0.1	47.3 ± 1.6	4.8 ± 0.2	13.4 ± 2.1	2.3 ± 1.2	73.4 ± 2.4	13.4 ± 2.3	381.6
	Florina	117.9 ± 8.7	66.9 ± 3.6	21.2 ± 1.2	3.5 ± 0.2	79.9 ± 3.3	10.6 ± 0.9	2.5 ± 0.2	0.6 ± 0.1	25.2 ± 1.3	4.6 ± 1.1	332.9
	Empire	77.8 ± 3.5	36.9 ± 3.1	13.1 ± 0.8	1.4 ± 0.3	46.7 ± 2.7	2.5 ± 0.1	2.4 ± 2.3	0.1 ± 0.0	24.3 ± 2.6	3.9 ± 0.4	209.1
Pear	**Fruits**											
	Hortensja	0.9 ± 0.2	0.3 ± 0.1	0.1 ± 0.0	nd	nd	nd	2.8 ± 0.6	nd	2.9 ± 0.1	0.8 ± 0.1	7.7
	Lukna	1.5 ± 0.3	2.2 ± 0.1	0.1 ± 0.0	nd	nd	nd	2.9 ± 0.3	nd	3.1 ± 0.1	0.8 ± 0.2	10.7
	Bonkreta Williamsa	1.7 ± 0.4	2.5 ± 0.1	0.1 ± 0.0	nd	nd	nd	4.7 ± 0.2	nd	4.3 ± 0.3	0.4 ± 0.1	13.6
	**Leaves**											
	Hortensja	143.1 ± 2.4	77.6 ± 2.6	27.7 ± 1.5	1.2 ± 0.3	94.2 ± 3.2	2.8 ± 0.3	3.1 ± 0.1	0.3 ± 0.0	40.5 ± 2.4	4.3 ± 0.2	394.7
	Lukna	243.9 ± 3.7	104.6 ± 7.4	48.0 ± 2.8	2.7 ± 0.3	113.4 ± 5.7	5.6 ± 0.8	5.1 ± 0.3	0.2 ± 0.0	54.0 ± 1.4	6.3 ± 0.1	583.9
	Bonkreta Williamsa	114.3 ± 5.1	94.6 ± 1.8	7.2 ± 0.5	0.9 ± 0.1	40.6 ± 3.8	2.9 ± 1.1	1.6 ± 0.2	0.4 ± 0.0	26.2 ± 1.0	5.6 ± 0.2	294.2
Quince	**Fruits**											
	Vranja	0.6 ± 0.1	4.0 ± 0.3	0.3 ± 0.0	nd	0.7 ± 0.1	0.2 ± 0.0	0.5 ± 0.0	nd	1.4 ± 0.3	0.0 ± 0.0	7.8
	ZM4	0.2 ± 0.0	1.0 ± 0.1	0.1 ± 0.0	nd	0.1 ± 0.1	0.2 ± 0.0	0.1 ± 0.0	nd	0.5 ± 0.1	0.0 ± 0.0	2.3
	CTM6	0.4 ± 0.1	1.5 ± 0.2	0.2 ± 0.0	nd	0.0 ± 0.0	0.1 ± 0.0	0.6 ± 0.0	nd	0.9 ± 0.2	0.0 ± 0.0	3.7
	**Leaves**											
	Vranja	226.3 ± 2.7	30.8 ± 2.1	42.5 ± 2.1	6.5 ± 1.2	134.9 ± 1.3	20.1 ± 1.1	3.3 ± 0.2	1.1 ± 0.2	26.9 ± 1.2	4.9 ± 0.2	497.4
	ZM4	346.7 ± 3.5	63.8 ± 1.6	69.7 ± 1.7	10.7 ± 1.3	171.6 ± 1.4	30.7 ± 1.4	5.5 ± 0.1	1.9 ± 0.1	64.3 ± 2.6	10.1 ± 1.0	775.0
	CTM6	271.7 ± 1.4	50.0 ± 2.8	54.6 ± 1.3	8.4 ± 0.9	134.4 ± 2.1	24.1 ± 1.2	4.3 ± 0.2	1.5 ± 0.5	50.4 ± 2.4	7.9 ± 0.6	607.3
Species	Apple	56.5b	29.9a	11.0b	1.3b	31.1b	0.0c	1.8a	0.3b	15.8b	2.3b	161.9b
	Pear	54.5b	44.2a	4.1b	0.0b	19.5b	3.7b	3.7a	0.2b	17.4b	3.0ab	146.6b
	Quince	169.7a	28.1a	35.2a	5.0a	84.9a	13.8a	3.5a	0.9a	34.6a	5.1a	380.5a
Fruits/leaves	Fruits	1.6b	1.9b	0.1b	0.0b	0.1b	0.04b	1.3b	0.0b	2.5b	0.2b	7.9b
	Leaves	185.5a	66.3a	33.4a	4.2a	95.6a	11.5a	4.6a	0.9a	42.7a	6.8a	451.4a
cultivars	Szampion	105.9a	42.1a	14.5bc	2.2a	32.3a	4.5a	7.8a	45.7a	45.8a	7.9a	265.3ab
	Florina	96.8a	38.2a	16.3abc	2.5a	48.5a	7.4a	2.4b	20.4bcd	20.4bcd	3.4b	236.5ab
	Empire	77.3a	23.2a	12.3bc	1.4a	32.0a	3.4a	2.4b	20.2bcd	20.2bcd	3.1b	175.5b
	Hortensja	110.7a	28.7a	26.5ab	2.7a	75.2a	7.3a	2.2b	26.8bc	26.8bc	2.9b	283.6ab
	Lukna	161.3a	43.1a	36.6a	3.4a	84.8a	8.7a	3.3b	33.7ab	33.7ab	3.9b	379.4a
	Bonkreta Williamsa	96.8a	38.2a	16.33abc	2.5a	48.5a	7.4a	2.4b	20.4bcd	20.4bcd	3.4b	236.5ab
	Vranja	37.0a	23.3a	2.9c	0.4a	30.6a	2.1a	1.5b	2.2d	2.2d	0.9b	100.9b
	ZM4	96.8a	38.2a	16.3abc	2.5a	48.5a	7.4a	2.4b	20.4bcd	20.4bcd	3.4b	236.5ab
	CTM6	59.5a	31.6a	8.8bc	1.4a	30.0a	4.0a	2.0b	13.6cd	13.6cd	2.3b	153.6b

Mean of 3 replications ± standard deviation followed by the same letter, within the same column were significantly different (p < 0.05) according to Tukey’s least significant differences test. Letters a,b,c,d, represent significance in content (p < 0.05).

*nd* not detected.

Regarding the carotenoid profile, 9-*cis* or 9-*cis'*-lutein and Σ of β-carotene with 9-*cis-*β-carotene were significantly higher for quince than for apple and pears. Among the carotenoids, 9-*cis* or 9-*cis'*-lutein predominated in both fruits and leaves but the content was differentiated by cultivar. In turn, leaves were up to 4 times richer in carotenoids content than fruits. As previously reported by Pop et al.^38^, content of carotenoids in sea buckthorn leaves depends on cultivar and equals 3.8–4.2 mg/100 g dw, and lutein was found in the highest concentration followed by β-carotene (0.9 and 0.7 mg/100 g dw, respectively). Lakshminarayana et al.^39^ reported the carotenoid content in some green leafy vegetables; it was 166.36 mg/100 g dw in dill and 238.62 mg/100 g dw in spinach.

The levels of chlorophylls were considerably higher relative to those of carotenoids, especially in leaves. The chlorophyll fraction was represented in leaves by chlorophylls and pheophytin as *a/b* and *a’/b’*. The content of chlorophylls in fruits was marginal, especially in apple and pear cultivars (> 10 mg/100 g dw). Regarding the chlorophyll profile, chlorophylls and pheophytin *a* were predominant because type *a* is a precursor for *b* type components. Pheophytin is synthesized from chlorophylls when naturally chelated magnesium in the chlorophyll macrocycle is readily substituted by hydrogen. Total chlorophyll concentration of fresh sea buckthorn leaves was 98.8 mg/100g^38^, which is lower than or comparable with our results. As reported by Ponder et al.^14^, raspberry leaves also contain chlorophylls in the range 6.7–9.6 mg/100 g fw, which significantly depends on cultivar.

Many epidemiological studies suggest that increased daily consumption of isoprenoid compounds decreases the risk of several degenerative and other diseases such as cardiovascular disease and skin cancer^14,38,39^.

#### Triterpenoids

The content of eleven triterpenoid compounds in the analyzed morphological parts of leaves and fruits of apple, pear, quince is shown in Table 3. Total triterpene for apple, pears and quince fruits and leaves was: 136.3–214.4 and 118.9–219.9 mg/100 g dw, 70.2–117.1 and 35.1–235.0 mg/100 g dw, and 65.2–152.3 and 91.3–226.4 mg/100 g dw, respectively. Overall, it has been observed that the leaves are an equal source of triterpenes as the analyzed fruits.

**Table 3:** Comparison of triterpene [mg/100 g dw] content in fruits and leaves of some selected species and cultivars of apple, pear, quince fruits and leaves.

Species	Cultivars	Tormentic acid	Maslinic acid	Pomolic acid	Corosolic acid	Betulinic acid	Oleanolic acid	Ursolic acid	Betulin	α-Boswellic acid	Erythrodiol	Uvaol	Total
Apple	**Fruits**												
	Szampion	0.6 ± 0.0	4.5 ± 0.1	3.7 ± 0.0	18.3 ± 0.9	3.2 ± 0.0	10.3 ± 1.2	4.5 ± 0.1	1.4 ± 0.1	2.6 ± 0.2	18.9 ± 2.3	2.2 ± 0.1	70.17
	Florina	3.1 ± 0.2	4.1 ± 0.1	4.1 ± 0.1	14.7 ± 1.2	5.7 ± 0.0	20.0 ± 2.1	4.0 ± 0.1	1.0 ± 0.0	0.1 ± 0.0	33.0 ± 1.4	10.1 ± 1.4	99.64
	Empire	0.5 ± 0.0	3.0 ± 0.2	4.2 ± 0.1	37.5 ± 1.2	9.3 ± 0.1	13.6 ± 0.4	16.7 ± 2.6	0.0 ± 0.0	0.0 ± 0.0	32.3 ± 2.5	0.0 ± 0.0	117.11
	**Leaves**												
	Szampion	6.8 ± 0.3	1.6 ± 0.0	1.6 ± 0.0	3.0 ± 0.1	5.2 ± 0.1	1.6 ± 0.4	1.6 ± 0.1	1.6 ± 0.2	0.2 ± 0.1	8.9 ± 1.3	2.9 ± 0.1	35.08
	Florina	9.9 ± 0.8	4.2 ± 0.1	3.5 ± 0.0	11.1 ± 0.3	12.2 ± 0.5	5.7 ± 0.1	11.7 ± 0.5	6.3 ± 0.1	0.6 ± 0.0	6.3 ± 0.7	7.5 ± 0.2	78.96
	Empire	49.1 ± 1.3	10.4 ± 0.2	13.6 ± 0.3	24.4 ± 1.2	21.2 ± 0.9	21.0 ± 0.7	42.2 ± 3.5	17.2 ± 0.5	10.0 ± 0.5	14.9 ± 1.6	11.0 ± 0.4	234.96
Pear	**Fruits**												
	Hortensja	4.6 ± 0.5	6.8 ± 0.1	11.1 ± 0.2	37.8 ± 2.4	9.0 ± 0.3	24.3 ± 0.9	52.2 ± 1.5	4.0 ± 0.1	0.8 ± 0.2	32.3 ± 2.5	6.9 ± 0.1	188.77
	Lukna	0.7 ± 0.1	8.2 ± 0.2	10.8 ± 0.3	37.6 ± 1.5	13.1 ± 0.1	32.9 ± 1.4	60.4 ± 3.6	2.4 ± 0.0	1.2 ± 0.1	37.0 ± 2.6	9.9 ± 0.5	214.36
	Bonkreta Williamsa	0.5 ± 0.0	3.3 ± 0.0	3.8 ± 0.1	23.6 ± 1.3	6.7 ± 0.3	18.6 ± 2.6	52.8 ± 2.1	2.1 ± 0.0	1.6 ± 0.2	14.6 ± 1.1	8.7 ± 0.2	136.31
	**Leaves**												
	Hortensja	11.5 ± 1.3	8.4 ± 0.2	7.4 ± 0.2	12.4 ± 0.6	17.0 ± 1.3	23.5 ± 2.1	56.7 ± 3.1	4.3 ± 0.2	4.1 ± 0.3	15.4 ± 1.3	12.9 ± 0.5	173.65
	Lukna	11.8 ± 1.0	8.5 ± 0.2	8.7 ± 0.1	14.1 ± 0.8	14.7 ± 0.8	9.2 ± 1.3	32.4 ± 1.7	2.0 ± 0.1	0.4 ± 0.1	8.4 ± 0.6	8.8 ± 0.1	118.93
	Bonkreta Williamsa	18.9 ± 0.7	15.5 ± 0.5	8.5 ± 0.3	25.4 ± 0.9	35.8 ± 2.1	21.9 ± 2.6	42.6 ± 1.9	17.4 ± 1.4	0.9 ± 0.1	15.7 ± 1.4	17.4 ± 1.9	219.94
Quince	**Fruits**												
	Vranja	3.6 ± 0.2	5.6 ± 0.1	10.3 ± 0.3	20.8 ± 1.1	8.0 ± 0.5	19.7 ± 2.5	49.8 ± 2.5	1.3 ± 0.2	1.6 ± 0.2	22.0 ± 2.1	9.4 ± 0.5	152.29
	ZM4	2.8 ± 0.1	3.6 ± 0.0	10.0 ± 0.2	25.7 ± 0.2	8.0 ± 0.7	6.8 ± 0.2	16.6 ± 1.4	0.8 ± 0.1	1.2 ± 0.3	27.5 ± 1.0	6.3 ± 0.2	108.97
	CTM6	1.4 ± 0.0	2.5 ± 0.0	4.3 ± 0.1	18.5 ± 1.2	6.0 ± 0.3	2.6 ± 0.6	6.4 ± 0.4	0.5 ± 0.0	0.3 ± 0.0	19.6 ± 1.5	3.3 ± 0.1	65.22
	**Leaves**												
	Vranja	2.9 ± 0.2	8.7 ± 0.4	5.4 ± 0.6	8.8 ± 0.4	9.9 ± 0.2	8.4 ± 0.4	24.8 ± 1.5	7.1 ± 0.3	1.2 ± 0.2	7.7 ± 0.6	17.8 ± 2.5	102.73
	ZM4	2.3 ± 0.1	7.4 ± 0.2	2.9 ± 0.1	8.9 ± 0.2	4.9 ± 0.1	4.8 ± 0.2	9.0 ± 0.8	6.1 ± 0.6	0.6 ± 0.1	13.6 ± 0.6	30.8 ± 2.7	91.26
	CTM6	2.7 ± 0.1	19.6 ± 1.3	6.8 ± 0.5	16.8 ± 0.5	13.0 ± 0.1	23.8 ± 1.5	28.6 ± 2.5	16.4 ± 1.4	2.3 ± 0.1	52.6 ± 2.6	43.8 ± 2.9	226.37
Species	Apple	8.3b	10.9a	10.0a	24.9a	16.9a	26.3a	52.6a	5.3b	1.7b	29.5a	15.1ab	187.5a
	Pear	26.8a	9.3a	9.2a	30.0a	18.2a	22.6a	35.7b	11.7a	5.7a	29.5a	11.3b	196.8a
	Quince	4.6b	8.1a	6.7a	16.4b	9.4b	11.1b	19.0c	6.5ab	1.6b	26.5a	24.2a	121.1b
Fruits/leaves	Fruits	5.1b	5.9b	8.0a	28.0a	9.6b	19.3a	33.1a	3.0b	1.8b	30.1a	9.1b	145.7a
	Leavs	16.0a	10.7a	7.5a	15.9b	16.8a	16.0a	31.6a	10.2a	3.0a	19.7b	19.7a	159.9a
Cultivars	Szampion	21.5a	9.7ab	10.8ab	29.5a	16.2ab	25.0a	48.9b	10.6abc	5.9a	33.8ab	18/9a	210.9a
	Florina	15.0a	8.4ab	9.6ab	27.1a	14.2ab	18.0ab	34.3b	6.5bc	3.1ab	26.9ab	14.5a	169.1ab
	Empire	23.1a	11.4ab	7.7abc	28.9a	24.4a	21.4ab	41.5b	16.2a	4.8a	25.1b	22.5a	207.8a
	Hortensja	8.1b	6.8b	5.0c	10.0b	6.1c	10.9b	13.9c	1.6d	0.7c	23.0b	15.9a	73.4c
	Lukna	1.5b	7.9b	6.1bc	12.2b	10.8bc	17.8ab	18.7c	3.8cd	1.1bc	28.7b	22.1a	109.9bc
	Bonkreta Williamsa	15.0a	8.4ab	9.6ab	27.1a	14.2ab	18.0ab	34.3b	6.5bc	3.1ab	26.9ab	14.5a	169.1ab
	Vranja	20.5a	12.1ab	12.7a	27.7a	19.6ab	30.4a	64.7a	9.5abc	4.7a	27.0b	13.9a	223.7a
	ZM4	15.0a	8.4ab	9.6ab	27.1a	14.2ab	18.0ab	34.3b	6.5bc	3.1ab	26.9ab	14.5a	169.1ab
	CTM6	19.2a	16.0a	10.4ab	30.6a	20.2ab	29.5a	44.9b	13.7ab	4.7a	48.2a	23.9a	241.9a

Mean of 3 replications ± standard deviation followed by the same letter, within the same column were significantly different (p < 0.05) according to Tukey’s least significant differences test. Letters a,b,c,d, represent significance in content (p < 0.05).

Generally, pear and apple had significantly (p < 0.05) higher content of triterpene compounds than quince. Oleanolic, corosolic, ursolic acids and erythrodiol prevailed in the apple fruits but ursolic and oleanolic acids were characteristic compounds for apple leaves. Corosolic and oleanolic acids and erythrodiol were predominant triterpenoid compounds for pear fruits and leaves. Ursolic and corosolic acids were major triterpenes for quince leaves and fruits, but in addition quince fruits were rich in erythrodiol and leaves were rich in uvaol. As previously mentioned, ursolic, oleanolic acids and uvaol are the main compounds identified in apple and cherry, oleanolic acid in grape berry and bilberry, olive, maslinic acid in olive but α-,β-,δ-amyrins was characterized for tomato^40^. There was no significant (p > 0.05) difference in total content of triterpene between leaves and fruits but leaves were significantly (p < 0.05) higher content of uvaol, α-boswellic, betulin, betulinic, maslinic and tormentic acids than fruits, where the major (p < 0.05) triterpenes were erythrodiol and corosolic acids. Szakiel et al.^41^ reported that mainly triterpene compound levels of bilberry leaves (oleanolic and ursolic acids) were significantly lower than those of berries.

A diet rich in triterpenoid compounds has been associated with some beneficial effects such as reduced incidence of many chronic diseases including cardiovascular, ischemic stroke, neurodegenerative disorders and aging and with numerous biological activities such as cytostatic, anti-inflammatory (COX inhibition), antibacterial and antiviral (including anti-HIV), anticarcinogenic or hypolipidemic and cholesterol-lowering^40^. The triterpenoids mainly accumulated in cuticular waxes and the differences in their concentrations depend on many factors, e.g. anatomical part of plant, climate and environmental conditions, humidity, and stage of maturity.

### Anti-oxidant, inhibitory of diabetic, obesity, cholinergic, lipo- and cyclooxygenase activity of apple, pear and quince fruits and leaves

#### Anti-oxidant capacity

**Figure 2:**
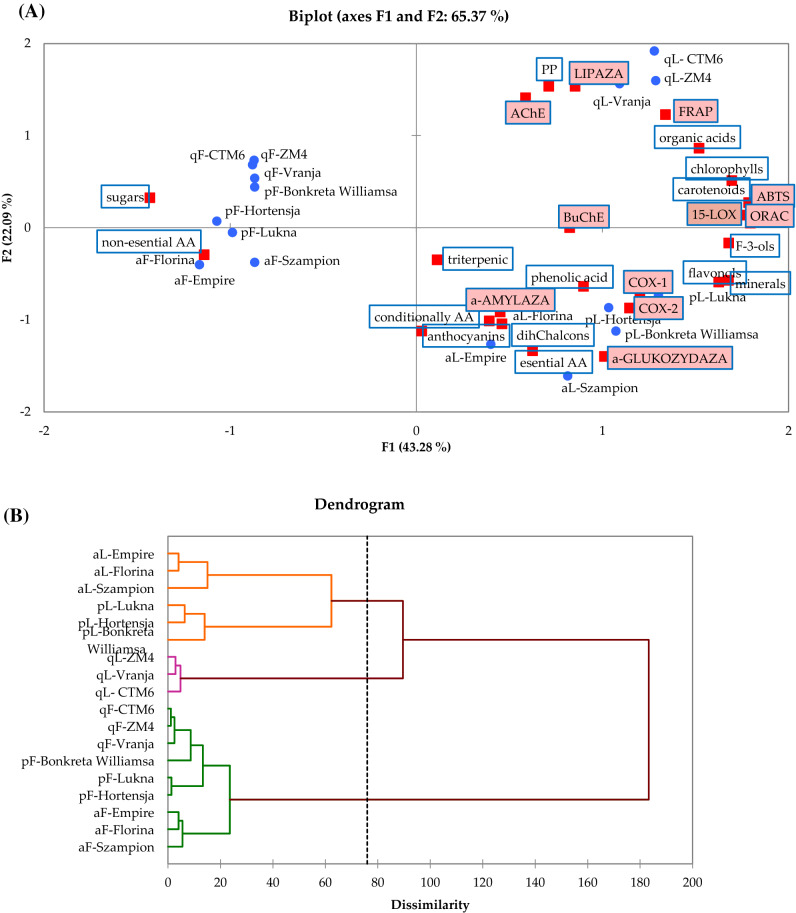
PCA biplot (A) showing the relationship and AHC dendrogram (B) based on dissimilarities among samples and chemical constituents with respect to apple (a), pear (p), quince (q) fruit (F) and leaves (L) cultivars.

The antioxidant capacity assayed in fruits vs. leaves of different cultivars of apple, pear and quince is presented in Table 4. For ABTS, FRAP and ORAC assay obtained results clearly indicate that all pome leaves showed significant differences between fruits and leaves. These values were 3.8, 3.5 and 3.0 and 1.2, 1.8 and 3.2 times for ABTS and FRAP higher than in the apple, pear and quince fruits of the respective cultivars, respectively. For ORAC assay these values were 10.1, 3.3 and 14.5 times higher than in the apple, pear and quince fruits of the respective cultivars, respectively. Higher antioxidant potential was determined in quince than in pears and apple, whether fruit or leaves were analyzed. Additionally, it was mentioned that cultivars were one factor influencing antioxidant capacity. It is known that the antioxidative potential depends on the content of bioactive components such as polyphenols, isoprenoid, vitamins or other molecules present in plants. As presented in Fig. 2, there was a significantly high correlation between chlorophylls, carotenoids, flavonols and ABTS, FRAP, ORAC (r^2^ > 0.808). Additionally, antioxidant capacity had an influence on polymeric procyanidins (FRAP as r^2^ = 0.789). Similar results were previously presented in the literature^10,14^.

**Table 4:** Anti-oxidant capacity and inhibition of hyperglycemic, obesity, inflammatory and cholinesterase enzyme, 15-lipooxygenase activity of apple, pear, quince fruits and leaves.

Species	Cultivars	Anti-oxidant capacity [mmol Trolox/100 g dw]			Enzyme inhibition [IC50; mg/ml]					Cholinesterase activity [%]		15-LOX [%]
		ABTS	FRAP	ORAC	Hyperglycemic		Obesity	COX-1	COX-2	AChE	BuChE	
					α-amylase	α-glucosidase	pancreatic lipase					
Apple	**Fruits**											
	Szampion	20.5 ± 1.2	16.6 ± 0.3	61.2 ± 1.3	8.4 ± 1.2	8.4 ± 1.2	2.3 ± 0.1	8.4 ± 1.2	40.5 ± 2.1	44.1 ± 1.2	78.0 ± 1.4	44.5 ± 2.1
	Florina	18.3 ± 1.0	17.9 ± 0.6	41.1 ± 1.4	4.9 ± 1.0	4.9 ± 1.0	1.5 ± 0.1	4.9 ± 1.0	12.0 ± 1.4	40.5 ± 1.3	71.6 ± 1.7	48.5 ± 1.4
	Empire	15.4 ± 0.5	13.8 ± 0.9	23.2 ± 1.1	7.8 ± 0.7	7.8 ± 0.7	2.2 ± 0.2	7.8 ± 0.7	28.0 ± 2.6	45.6 ± 1.0	75.0 ± 1.1	47.1 ± 1.1
	**Leaves**											
	Szampion	51.6 ± 1.3	20.7 ± 0.4	492.3 ± 2.6	5.5 ± 0.9	5.5 ± 0.9	1.1 ± 0.1	5.5 ± 0.9	6.1 ± 0.2	51.9 ± 1.0	81.6 ± 1.8	81.3 ± 2.5
	Florina	55.8 ± 1.1	17.1 ± 0.3	412.2 ± 2.6	4.5 ± 0.4	4.5 ± 0.4	1.0 ± 0.1	4.5 ± 0.4	4.9 ± 0.3	56.4 ± 1.4	77.9 ± 1.2	77.8 ± 2.4
	Empire	43.1 ± 0.4	20.0 ± 0.8	364.8 ± 3.5	6.0 ± 0.2	6.0 ± 0.2	0.5 ± 0.0	6.0 ± 0.2	5.6 ± 0.2	56.6 ± 0.9	77.2 ± 1.9	82.7 ± 1.8
Pear	**Fruits**											
	Hortensja	18.1 ± 0.5	16.1 ± 0.5	91.3 ± 2.1	35.2 ± 1.1	35.2 ± 1.1	0.1 ± 0.0	35.2 ± 1.1	10.2 ± 0.5	50.1 ± 0.5	9.4 ± 0.4	53.7 ± 1.2
	Lukna	23.2 ± 0.6	21.3 ± 0.2	145.4 ± 2.5	30.2 ± 1.3	30.2 ± 1.3	0.8 ± 0.0	30.2 ± 1.3	36.5 ± 0.7	39.9 ± 0.2	16.7 ± 1.7	49.8 ± 2.1
	Bonkreta Williamsa	30.6 ± 0.9	28.8 ± 0.7	150.0 ± 2.6	34.2 ± 1.2	34.2 ± 1.2	0.2 ± 0.0	34.2 ± 1.2	70.4 ± 1.1	79.1 ± 1.3	74.8 ± 2.4	40.2 ± 2.5
	**Leaves**											
	Hortensja	80.8 ± 1.4	37.6 ± 0.5	439.8 ± 3.1	4.3 ± 0.6	4.3 ± 0.6	0.9 ± 0.1	4.3 ± 0.6	7.7 ± 0.6	40.7 ± 0.3	66.1 ± 4.1	83.2 ± 1.1
	Lukna	86.3 ± 1.0	41.7 ± 0.3	499.1 ± 2.6	4.1 ± 0.3	4.1 ± 0.3	1.3 ± 0.3	4.1 ± 0.3	11.5 ± 1.1	50.9 ± 0.2	58.3 ± 2.5	75.5 ± 1.5
	Bonkreta Williamsa	83.1 ± 1.1	39.3 ± 0.6	338.8 ± 4.2	4.6 ± 1.1	4.6 ± 1.1	1.0 ± 0.1	4.6 ± 1.1	9.1 ± 0.7	46.3 ± 0.3	57.3 ± 3.2	82.7 ± 2.1
Quince	**Fruits**											
	Vranja	22.9 ± 0.4	20.3 ± 0.6	21.7 ± 1.2	9.3 ± 0.6	9.3 ± 0.6	15.0 ± 0.6	9.3 ± 0.6	17.3 ± 1.2	56.7 ± 0.3	48.3 ± 1.1	57.9 ± 2.5
	ZM4	31.1 ± 0.1	24.2 ± 0.5	49.7 ± 2.5	10.5 ± 1.4	10.5 ± 1.4	17.2 ± 0.3	10.5 ± 1.4	12.1 ± 1.1	68.3 ± 0.5	42.3 ± 2.6	60.1 ± 2.9
	CTM6	30.6 ± 0.8	26.5 ± 0.3	39.4 ± 2.7	7.9 ± 0.3	7.9 ± 0.3	22.1 ± 0.3	7.9 ± 0.3	17.1 ± 1.5	60.9 ± 1.2	62.8 ± 3.1	57.3 ± 0.9
	**Leaves**											
	Vranja	81.0 ± 0.9	77.2 ± 1.5	464.0 ± 2.1	1.7 ± 0.5	1.7 ± 0.5	4.4 ± 0.1	1.7 ± 0.5	0.9 ± 0.2	72.4 ± 1.1	77.9 ± 0.7	86.8 ± 1.5
	ZM4	82.3 ± 1.1	74.5 ± 1.2	588.9 ± 1.1	3.3 ± 0.2	3.3 ± 0.2	4.7 ± 0.2	3.3 ± 0.2	3.5 ± 0.1	82.0 ± 0.7	78.0 ± 1.1	91.0 ± 1.5
	CTM6	88.0 ± 1.4	74.1 ± 1.0	553.4 ± 2.6	2.4 ± 0.1	2.4 ± 0.1	6.1 ± 0.1	2.4 ± 0.1	2.7 ± 0.3	86.8 ± 0.9	82.7 ± 2.1	93.2 ± 2.1
Species	Apple	34.1b	17.7c	232.5b	6.2a	6.2a	1.4a	6.2a	16.2a	49.2b	76.9a	63.7b
	Pear	53.7a	30.8b	277.4ab	18.8b	18.8b	0.7a	18.8b	24.2b	51.2b	47.1c	64.2b
	Quince	56.0a	49.5a	286.2a	5.9a	5.9a	11.6b	5.9a	8.9a	71.2a	65.3b	74.4a
Fruits/leaves	Fruits	23.4b	20.6b	69.2b	16.5b	16.5b	6.8b	16.5b	27.1b	53.9b	53.2b	51.0b
	Leaves	72.4a	44.7a	461.5a	4.0a	4.0a	2.3b	4.0a	5.8a	60.4a	73.0a	83.8a
Cultivars	Szampion	36.0bc	18.6b	276.8ab	6.9a	6.9a	1.7a	6.9a	23.3ab	48.0bc	79.8a	62.9c
	Florina	37.1bc	17.5b	226.7ab	4.7a	4.7a	1.2a	4.7a	16.8a	48.5bc	74.7a	63.1c
	Empire	29.2c	16.9b	194.0b	6.9a	6.9a	1.4a	6.9a	16.8a	51.1bc	76.1a	64.9bc
	Hortensja	49.5ab	26.9b	265.5ab	19.7b	19.7b	0.5a	19.7b	9.0a	45.4c	37.8b	58.5abc
	Lukna	54.8a	31.5ab	322.2a	17.1ab	17.1ab	1.0a	17.1ab	24.0ab	45.4c	37.5b	62.7c
	Bonkreta Williamsa	56.9a	34.1ab	244.4ab	19.4b	19.4b	0.6a	19.4b	39.7b	62.7abc	66.1a	61.5c
	Vranja	51.9a	48.7a	242.9ab	5.5a	5.5a	9.7b	5.5a	9.1a	64.6ab	63.1a	72.3ab
	ZM4	56.7a	49.4a	319.3a	6.9a	6.9a	11.0b	6.9a	7.8a	75.1a	60.1ab	75.6a
	CTM6	59.3a	50.3a	296.4ab	5.2a	5.2a	14.1b	5.2a	9.9a	73.8a	72.7a	75.3a

Mean of 3 replications ± standard deviation followed by the same letter, within the same column were significantly different (p < 0.05) according to Tukey’s least significant differences test. Letters a,b,c,d, represent significance in content (p < 0.05).

#### Inhibitory of hyperglycemic and obesity activity

Pome fruits and leaves were also analyzed as sources of bioactive substances with hyperglycemic and obesity activities. The α-amylase activity was significantly dependent on type of pome species where quince and apple presented greater activity than pear. Quince leaves are more active inhibitors of α-amylase than other samples, especially pears. Contrary to α-amylase activity leaves of pome species showed significantly higher α-glucosidase and lipase activity than fruits. Higher potential of α-glucosidase inhibitory was shown by quince leaves and fruits than apple and pears. Higher lipase inhibitory potential was presented by pear and apple leaves and fruits than quince. Cultivar had no significant influence on α-amylase, α-glucosidase or lipase activity.

Figure 2A presents a high, significant correlation between α-amylase and phenolic acid (r^2^ = 0.827) where other compounds play a minor impact. There was a correlation between α-glucosidase and non-essential amino acids (r = 0.902) and between lipase and polyphenols or sugars (r^2^ = 0.617 and 0.532, respectively). The enzymes α-amylase and α-glucosidase are responsible for breakdown of complex saccharides before absorbates during digestion and their active inhibition is important for people with type 2 diabetes mellitus. Pancreatic lipase enzyme is responsible for the breakdown of dietary fats to become absorbable in the intestinal lumen as monoacylglycerols and free fatty acids and their inhibition is important for people for obesity prevention and weight management^23,30,42,43^. Similar results were previously presented by Spinola et al.^42^, whereas leaves of *Elaeagnus umbellata* and *Sambucus lanceolata* present significantly higher inhibition than their berries against α-glucosidase and pancreatic lipase enzyme but opposite for α-amylase.

#### Cholinergic enzyme activity

Significant differences concerning the inhibitory activity towards AChE and BuChE activity were noted between all the pome fruits and leaves (Table 4). Our findings showed higher inhibition of leaves against AChE and BuChE activity than fruits. Inhibitory effects in the tested leaves were highest (p < 0.05) for quince > pear > apple and were 71.7, 56.9, 51.3% for AChE and 72.9, 66.4 78.2% for BuChE, respectively. Cultivar as a factor had no significant influence on these activities. BuChE and AChE are key enzymes in the breakdown of an important neurotransmitter, acetylcholine, playing a key role in pathogeneses of Alzheimer’s disease, currently one of the most prevalent neurodegenerative disorders. Therefore, these results present new promising sources of cholinergic inhibitory. AChE activity was only highly correlated with polymeric procyanidins and organic acids (r^2^ = 0.719 and 0.622, respectively). The other compounds present weak influences on AChE and BuChE activity (r^2^ < 0.650–0.413).

#### Anti-inflammatory activity

The anti-inflammatory effects of pome fruits and leaves were evaluated based on their ability to inhibit the activities of 15-LOX (as %), COX-1 and COX-2 (as IC_50_), as presented in Table 4. LOX and COX are important enzymes in lipid metabolism and oxidation of some linoleic and arachidonic acids for their corresponding metabolites, *cis*-, *trans*-conjugated hydroperoxides and prostaglandin, respectively^44^. Fruits show lower inflammatory activity to inhibit 15-LOX, COX-1 and COX-2. The results of 15-LOX inhibition clearly showed the great variation of obtained values between tested fruits vs. leaves (p ≤ 0.05). The highest potential was exhibited for quince leaves vs. fruits (approx. 95.4 vs. 58.4%), pear (approx. 80.5 vs. 47.9%), and apple (approx. 80.6 vs. 45.7%). Considering results obtained after COX analysis, significant differences were noted for leaves and fruits. Our present results of the COX inhibition studies focus on the importance of selected botanicals as an important resource for the isolation and identification of new COX-2 selective anti-inflammatory agents. Leaves of all pome species showed higher activity, especially pear for COX-1, and apple and quince for COX-2. The 15-LOX activity was modulated between flavan-3-ols, flavonols, chlorophylls, carotenoids, minerals and organic acids (r^2^ = 0.768, 0.745, 0.867, 0.883, 0.855, 0.845, respectively). In previous research extracts of *Jamaican Rubus* spp. demonstrated moderate COX inhibitory activity (27.5–33.1%) at 100 mg/mL^45^. These results indicated that leaves have high anti-inflammatory effects, which can be presumably related to bioactive compounds present in leaves and fruits. Flavonoids inhibit biosynthesis of prostaglandins (the end products of the COX and lipoxygenase pathways), which act as secondary messengers and are involved in various immunologic activities^44^. LOX and COX play an important role in several inflammatory diseases such as cancer, bronchial asthma, osteoporosis, allergic, atherosclerosis, and arthritis^44^.

#### Principal component analysis (PCA) and hierarchical clustering analysis (HCA)

To investigate and better understand their variation and the relationship between biological activity, nutritional and bioactive compounds of apple, pear and quince fruits vs. leaves, PCA and HCA were performed (Fig. 2A, B). The first two PCA account for about 65.37% of the total variance, which showed that nearly all the data variation can be explained by PC1–PC2. As can be seen, pome leaves samples are found on the right half of the PCA graphic, whereas pome fruits appear mainly on the negative half of the graphic.

Pome leaves were associated with some nutritional compounds (minerals, amino acids (conditional and nonconditional), organic acids) and bioactive compounds (polyphenols, carotenoids, chlorophylls, triterpenic) whereas fruits were associated only with high content of sugars and non-essential amino acids. Additionally, the PCA relationship indicates that leaves were associated with all biologically activity. Quince leaves are rich in polymeric procyanidins, organic acids, chlorophylls, carotenoids and they primarily associated with AChE and pancreatic lipase. Apple and pear leaves are rich in triterpenic, phenolics (phenolic acids, flavonols, anthocyanins, dihydrochalcones, flavan-3-ols), essential and non-essential amino acids, and minerals primarily associated with α-amylase, α-glucosidase, COX-1 and COX-2. All investigated leaves showed high antioxidant potential as FRAP, ABTS, ORAC and 15-LOX activity.

Similar to PCA, HCA (Fig. 2B) presented two separate clusters for leaves vs. fruits. Inspection of the groups showed that the quince clustered separately from apple and pears, being characterized by high concentrations of polymeric procyanidins, organic acids, chlorophylls and carotenoids.

## Conclusions

Various plant materials and their morphological parts are currently being investigated as potential sources of bioactive compounds. This study demonstrated significant differences between leaves and fruits of pome species—apple, pear, and quince—in the content of both bioactive and some nutritional compounds and biological activity. Leaves of pome species were richer in isoprenoid such as chlorophylls and polyphenolics. The dominant fraction for quince, pear, and apple fruits comprised polymeric procyanidins. In quince and pear leaves flavan-3-ols and flavonols dominated, while in apple the polyphenols were represented by dihydrochalcones > flavonols ~ flavan-3-ols. The differences in the content of triterpene in the analyzed morphological parts occur since these compounds are mainly accumulated in the waxy layer of the plants and therefore fruits are richer in triterpene than leaves. Leaves are excellent sources of amino acids and mineral compounds, especially Ca, Mg, Fe, and K. They are characterized by a high content of organic acids and low content of sugars compared to fruits of pome species. Leaves of apples and pears showed the most effective inhibition of COX-1, COX-2, α-amylase, and α-glucosidase but quince leaves showed the most effective inhibition of pancreatic lipase, AChE and BuChE, 15-LOX, and antioxidant capacity, which were particularly correlated with bioactive compounds.

Thus, these results indicate that leaves of pome species are attractive, unconventional sources of bioactive compounds for preparing some nutraceutical foods for use in the prevention of selected disease entities, if they will be acceptance by the consumers. Additionally, leaves of pome species can also be used as valuable sources for the pharmaceutical and cosmetic industries.

## Materials and methods

### Plant materials

Nine samples of pome fruits (approx. 1 kg) and leaves (approx. 0.5 kg) of apple (*Malus domestica* Brok. cultivars Szampion, Florina, Empire), pears (*Pyrus communis* L., cultivars Hortensja, Lukna, Borketa Williamsa) and quince (*Cydonia oblonga* Mill., cultivars Vranja, ZM4, CTM6) were collected during September 2019 at the Research Station for Cultivar Testing in Zybiszów near Wrocław (51°3′51.11′′N, 16°54′43.56′′E). The fruits were harvested optimally ripe based on extracts, flavor, color, and structure. At the same time leaves were collected. All sample were collected from three batches and they are the sources of three replications for all analyses and for the lyophilization step, fruits were cut and leaves were frozen in liquid nitrogen (Christ Alpha 2–4; Braun Biotech Int., Melsungen, Germany), ground in a laboratory mill (IKA, A11; Darmstadt, Germany) and kept at -20 °C for 7 days until analysis.

### Organic acid and sugar determination

The organic acid and sugar content was determined using the method proposed previously by Wojdyło et al.^30^ by HPLC–PDA (Waters Co.; Milford, CT, USA) and HPLC-ELSD (PL-ELS 1000; Merck; Hitachi, Japan), respectively. The sample (approx. 3 g of fruits and 1 g of leaves) mixed with distilled water, sonicated (Sonic 6D; Polsonic, Warsaw, Poland) for 15 min and boiled for 30 min, finally sample was centrifuged (MPW-55; Warsaw, Poland) at 12,000x*g* for 10 min at 4 °C. The supernatant (2.5 mL) was applied onto the Sep-Pak C-18 (1 g, Millipore Waters, Milford, MA, USA) and finally eluted by water to Eppendorf tubes. The extract before analysis was filtered through 0.20 μm hydrophilic PTFE membrane (Millex Simplicity Filter; Merck, Germany). All samples were assayed in triplicate repetition. Results expressed as g per 100 g dry weight (dw).

### Mineral determination

The mineral content was determined using the method proposed previously by Carbonell-Barrachina et al.^46^*.* A microwave digestion block system (Multiwave GO, Anton Para; Graz, Austria) was used for sample mineralization. The sample (approx. 1.0 and 0.50 g of fruits and leaves, respectively) was treated with 10 mL of 65% (*w/v*) HNO_3_ in Pyrex tubes, placed in the digestion block, and heated at 15 min for 200 °C, 25 min at 200 °C, and 10 min for cooling to 20 °C. Solutions were left to cool, transferred to a volumetric flask, and diluted with ultrahigh-purity deionized water. Determination of some minerals (Na, K, Mg, Ca, Fe, and Cu) in previously acid-mineralized samples was performed with an Atomic Absorption Spectrophotometer AA-7000 (Shimadzu; Kyoto, Japan). The instrumental conditions used for mineral determination were as follows: P at 213 nm, Fe at 248 nm, Mg at 285 nm, Cu at 324 nm, Ca at 422 nm, Na at 589 nm, and K at 766 nm and acetylene flame with a fuel flow rate of 0.9 L/min. Calibration curves were used for the quantification of minerals and showed good linearity (*R*^*2*^ = 0.997). The analysis of minerals was run in triplicate.

### Preparation and estimation of polyphenols, isoprenoids, triterpenic and amino acids

Analysis of polyphenols, isoprenoids, triterpenic and amino acids of fruits and leaves was carried out using an Acquity UPLC system (Waters Corp., Milford, MA, USA) equipped with a photodiode (PDA) detector. Analysis of polymeric procyanidins was carried a UPLC equipped with a fluorescence detector (FL) Acquity system (Waters Corp., Waters Corp.; Ireland). All samples were assayed n = 3, and the results were expressed as mg per 100 g of dw.

### Polyphenols

Polyphenols extraction and analysis was determined using the method proposed previously by Wojdyło et al.^30^. For extraction a MeOH/H_2_O/ascorbic acid (30:68:1, *v/v/m*) with 1% of 37% hydrochloric acid mixture were used. For polyphenolic analysis 5 μL of sample was injection by autosampler on BEH C18 column (2.1 × 100 mm, 1.7 μm; Waters Corp.; Dublin, Ireland) at 30 °C with gradient elution of solvent A (2.0% formic acid) and solvent B (acetonitrile) at a flow rate of 0.42 mL/min for a duration of 15 min. Spectra (λ) and retention times (Rt) were compared with those of pure standards for flavan-3-ols and dihydrochalcones at 280 nm (e.g. (−)-epicatechin, (+)-catechin, procyanidin B1, pholoridzin, phloretin), for phenolic acid at 320 nm (e.g. chlorogenic, neochlorogenic and 3,5-di-caffeoylquinic acids), for flavonols at 360 nm (e.g. quercetin- and kaempferol-3-*O*-glucoside), for anthocyanins at 520 nm (cyanidin-3-*O*-glucoside).

### Polymeric procyanidins

Polymeric procyanidins were determined by the phloroglucinolysis method as proposed previously by Wojdyło et al.^30^. For polymeric procyanidins 5 μL of sample was injection by autosampler on BEH C18 RP column (2.1 × 5 mm, 1.7 μm; Waters Corporation, Milford, MA, USA) at 15 °C with gradient elution of solvent A (2.5% acetic acid) and solvent B (acetonitrile), at a flow rate of 0.42 mL/min for a duration of 10 min. The detection was recorded at an emission wavelength of 360 nm and an excitation wavelength of 278 nm. The calibration curves were established using (+)-catechin, (−)-epicatechin, and procyanidin B1 after the phloroglucinol reaction as (+)-catechin and (−)-epicatechinphloroglucinol adduct standards.

### Isoprenoids

Isoprenoids as carotenoids and chlorophylls extraction and analysis were determined using the method proposed previously by Wojdyło et al.^30^. For extraction a hexane:acetone:methanol (2:1:1, *v*/*v*/*v*) mixture were used in the dark. For analysis 10 μL of sample was injection by autosampler on BEH RP C18 column (2.1 × 100 mm, 1.7 μm; Waters Corp.; Dublin, Ireland) at 32 °C with gradient elution of solvent A (0.1% formic acid) and solvent B (acetonitrile:methanol, 7:3, *v*/*v*) at a flow rate of 0.5 mL/min for a duration of 16.60 min. Spectra (λ) and retention times (Rt) were compared with those of pure standards for carotenoids (lutein, zeaxanthin, and ß-carotene) at 450 nm and for chlorophylls (chlorophylls *a*, pheophytin *a*) at 430 nm.

### Amino acids

Amino acids extraction and analysis were determined using the method proposed previously by Collado-González et al.^47^. For extraction methanol/water (1:1, *v*/*v*) mixture were incubated for 10 min at 55 °C. For amino acids analysis 3 μL was injection by autosampler on AccQ Tag Ultra BEH column (2.1 × 100 mm, 1.7 μm; Waters Corp.; Ireland) at 50 °C with gradient elution of solvent A mix of acetonitrile:formic acid:ammonium acetate (10:6:84, *v*/*v*/*v*) and solvent B (acetonitrile:formic acid, 99.9:0.1, *v*/*v*) at a flow rate of 0.5 mL/min for a duration of 15 min. Retention times (Rt) were compared with those of pure standards for amino acids at 260 nm.

### Triterpene

For extraction of triterpene 0.1–0.2 g samples were mixed with hexane/ethyl acetate (1:1, *v/v*) and sonicated (30 min at 60 °C) mixture were incubated for 12 h at 4 °C and collected supernatant extraction was repeated by addition chloroform:dichlorometane (1:1, *v/v*). Collected solvent were finally evaporated to dryness (XCV–5400 XcelVap Evaporation System, Horizon Technology, Inc.; Salem, USA) and diluted in MeOH. For triterpene analysis 3 μL was injection by autosampler on Zorbax Eclipse PAH column (2.1 × 150 mm 3.5-micron; Agilent Technology, St. Clara, USA) at 30 °C with gradient elution of solvent A as acetonitrile and solvent B as H_2_O at a flow rate of 0.25 mL/min for a duration of 30 min. Retention times (Rt) were compared with those of pure standards for triterpenic at 210 nm as: tormentic, maslinic, pomolic, carosolic, betulinic, oleanolic, ursolic, α-boswallic acids and betulin, eryhrodiol, uvaol.

### Biological in vitro activity and antioxidant capacity

#### *Extraction for biological *in vitro* analysis*

For the determination of biological in vitro activity, the lyophilized powdered sample (approx. 1 g for fruits and 0.5 g for leaves) was taken, and 7 mL of methanol:water: 37% hydrochloric acid (80:19:1, *v/v/w*) was added to each sample, sonicated (Sonic 6D; Polsonic, Warsaw, Poland) for 20 min and being incubated overnight (4 °C). After 24 h the slurry was centrifuged (MPW-55; Warsaw, Poland) at 19,000×*g* at 4 °C for 10 min to obtain extract for all biological in vitro analysis.

All tests were performed in triplicate using a microplate reader Synergy H1 (BioTek, Winooski, VT, USA).

#### Inhibitory of hyperglycemic (α-glucosidase and α-amylase) and obesity (pancreatic lipase) enzyme

Analysis of: α-amylase, α-glucosidase and pancreatic lipase were determined using the method proposed previously by Wojdyło et al.^30^.

The α-amylase inhibitory activity is based on a result of a reaction of iodine in potassium iodide with the remaining starch after enzymatic hydrolysis after incubation at 37 °C and absorbance was measured at 600 nm. The analysis of α-glucosidase inhibitory activity consists of the reaction of the enzyme with a β-D-glucosidase substrate measured at 405 nm. As in the above analysis, the reference samples contained buffer instead of enzymes and for above analysis the acarbose was included as a positive control.

The analysis of pancreatic lipase inhibitory activity is based on the amount of *p*-nitrophenol formed from *p*-nitrophenyl acetate. Basic samples with enzyme and substrate incubated at 37 °C and absorbance was measured at 400 nm. The reference samples contained buffer instead of enzymes and for above analysis the orlistat was used as a positive control.

The results of α-amylase, α-glucosidase, and pancreatic lipase activity are presented as the amount of the sample that is able to reduce enzyme activity by 50% as IC_50_ in mg/mL.

#### Inhibitory of 15-lipoxygenase assay

The 15-lipooxygenase inhibitory assay were determined using the method proposed previously by^23^ as on a results of a reaction of extract, enzyme and linoleic acid incubated at 37 °C for 20 min. This method defined the increase on absorbance at 210 nm as a result of the formation of conjugate double bonds in the linoleic acid hydroperoxide. Reference samples contained Tris–HCl buffer instead of the enzyme. The results were expressed as a % of inhibition.

#### Anti-inflammatory activity as cyclooxygenase (COX-1 and COX-2) assay

Anti-inflammatory activity as COX-1 and COX-2 inhibition enzyme was determined according using a protocol described in COX Inhibitor Screening Assay Kit (Cayman, No. 560131). The results of COX-1 and COX-2 are presented as the amount of the sample that is able to reduce enzyme activity by 50% as IC_50_ in mg/mL.

#### Inhibitory of cholinesterase activity (acetylcholinesterase (AChE) and butylcholinesterase (BuChE)

Analysis of: AChE and BuChE were determined using the method proposed previously by Wojdyło et al.^30^. The substrate of acetylcholine iodine and butylcholine chloride is hydrolyzed by the enzyme to thiocholine, which reacts with 5,5'-dithiobis-(2-nitrobenzoic acid) to produce 2-nitrobenzoate-5-mercaptothiocholine and 5-thio-2-nitrobenzoate detected at 405 nm. The results are expressed as % of inhibition.

#### Antioxidant capacity by FRAP, ABTS^·+^ and ORAC assay

The FRAP (involves determining the ability to reduce Fe^+3^ ions), ABTS (based on measuring the decrease in the color intensity inversely proportional to the antioxidant content) and ORAC assays (decrease in fluorescence caused by oxidation of a fluorescent substance under the influence of free radicals) were determined using the method proposed previously by^48,49^ and ^50^, respectively.

The 2,4,6-Tris(2-pyridyl)-s-triazine (TPTZ) diluted in HCl and FeCl_3_ × 6H_2_O were mixed with sample extract and after 10 min of reaction the absorption at the 593 nm was measured.

The 2,2′-azine-bis-(3-ethylene-benzothiazoline-6-sulfonic acid (ABTS) were mixed with sample extract and after 6 min of reaction the absorption at the 734 nm was measured.

The 2,2’-azobis(2-amidinopropane)dihydrochloride was added to sample extract, phosphate buffer, and fluorescein (incubated at 37 °C) were mixed and measured performed every 5 min at an excitation and an emission wavelength 493 and 515 nm, respectively. The blank was a phosphate buffer.

The results for FRAP and ABTS were calculated based on the calibration curve (R^2^ = 0.9950) for Trolox concentrations 0.050 to 0.900 mM and 0.100 to 0.900 mM, respectively. The results of ORAC assay were obtained by comparing the surface under the fluorescence decrease curves over time with the surface for pure Trolox solutions (12.5, 25.0, 50.0, and 75.0 μM). The FRAP, ABTS, and ORAC results were expressed in mmol TE (Trolox)/100 g sample.

### Statistical analysis

Results are presented as mean values of n = 3 for each cultivar analysis ± standard deviation. Principal Components Analysis (PCA) and Hierarchical Clustering Analysis (HCA) were performed on *XLSTAT* 2017 (Addinsoft, New York, NY, USA). One-way analysis of variance (ANOVA) by Tukey’s test were performed.

### Ethic statement

Research did not include any human subjects and animal experiments. All the authors declare that plants were used in accordance with relevant guidelines and regulations. The authors had permission to collect all plant samples used in the study.
